# The Importance of Sensory Lexicons for Research and Development of Food Products

**DOI:** 10.3390/foods8010027

**Published:** 2019-01-15

**Authors:** Suntaree Suwonsichon

**Affiliations:** Kasetsart University Sensory and Consumer Research Center, Department of Product Development, Faculty of Agro-Industry, Kasetsart University, Bangkok 10900, Thailand

**Keywords:** sensory, descriptive analysis, lexicon, food, product development, shelf life, quality control, product improvement, plant breeding

## Abstract

A lexicon is a set of standardized vocabularies developed by highly trained panelists for describing a wide array of sensory attributes present in a product. A number of lexicons have been developed to document and describe sensory perception of a variety of food categories.The current review provides examples of recently developed sensory lexicons for fruits and vegetables; grains and nuts; beverages; bakery, dairy, soy and meat products; and foods for animals. Applications of sensory lexicons as an effective communication tool and a guidance tool for new product development processes, quality control, product improvement, measuring changes during product shelf life, and breeding new plant cultivars are also discussed and demonstrated through research in the field.

## 1. Introduction

Descriptive sensory analysis is the most powerful method for capturing a product’s characteristics in terms of their perceived attributes and intensities. This method has been used extensively to characterize the sensory characteristics of various food products [1]. A lexicon is a set of standardized vocabularies developed and used by panelists who are highly trained for describing a wide array of sensory attributes present in a product. Lexicon development is one of the crucial steps in descriptive sensory analysis. To develop a lexicon, the panelists evaluate samples that, as much as possible, represent the entire product space, generate terms that describe the samples, define the terms, develop standardized evaluation procedures, select references that clarify the terms, review samples to further train the panelists, and then finalize those terms [2]. In most cases, panelists also assign a score for each reference standard to anchor an attribute scale. Terms listed in the lexicon must be extensive and complete, non-hedonic, singular (not integrated), and non-redundant, and also must capture all product differences [3]. Previously, Lawless and Civille [2] and Drake and Civille [4] outlined important considerations for lexicon development and reviewed a number of food lexicons published before the year 2013. 

A sensory lexicon plays an important role as an effective communication tool among diverse audiences such as sensory panels, sensory scientists, product developers, marketing professionals, and suppliers who may have different understandings of the same sensory attribute due to differences in perception, background knowledge, and culture. In addition, carrying out descriptive sensory analysis using a lexicon with well-defined and referenced descriptors and standardized evaluation procedures provide accurate and repeatable information about sensory qualities of food products that could be used as a guidance tool for various activities in the research and development of food products, including new product development, quality control, product improvement, measuring changes during product shelf-life, and breeding new plant cultivars. 

The aims of this article were to: (1) review recently developed food lexicons, and (2) discuss and exemplify how the lexicons could be used to strengthen research and development of food products. 

## 2. Recent Lexicons for Foods

A number of lexicons have been developed to describe sensory characteristics of a variety of food products, including: fruits and vegetables; grains and nuts; beverages; bakery, dairy, soy and meat products; and foods for animals. Some lexicons further explored complex sensory attributes (e.g., smoky) that may have a different character depending on the food product. Examples of recent lexicons of various food categories published mostly during the year 2013–2018 are as follows. All of them were developed by trained panelists using a descriptive sensory analysis approach. Lexicons may be developed as part of the process to evaluate a set of products, and may make that lexicon particularly useful for other researcher’s projects if the tested product set was large and encompassed a wide range of samples. Lexicons may also be developed as a stand-alone project in an attempt to provide a starting point for many researchers to have a common ground on which to build studies that can be compared. Both types of lexicon are included in this review. Papers that included terminology or groups of words developed by consumers, minimally trained panelists, or that contained only a few terms or included liking or quality characteristics were not included in this review.

### 2.1. Fruits and Vegetables

Recent examples of lexicons for fruits, vegetables, and related products include those for apple, pomelo, peach, blackberry, strawberry, pomegranate, mango puŕee, sweet tamarind, and cabbage kimchi. Corollaro et al. [5] defined and referenced 15 terms to describe sensory differences, mainly texture properties, among apple cultivars and perceivable changes of the fruit during refrigerated storage. More recently, Bowen et al. [6] examined 78 apple cultivars and developed a lexicon for describing the diversity in flavor and texture characteristics of the fruit among cultivars. For pomelo, 30 descriptors were developed to distinguish between fruit of different cultivars at various fresh-cut storage periods [7]. Belisle et al. [8] established a lexicon containing aroma, flavor, and texture attributes that described differences among cultivars and stages of ripeness of peach (mature, under-ripe, over-ripe). Du et al. [9] developed a lexicon for describing aroma characteristics of blackberry of seven commercial cultivars and two newly-bred cultivars. Oliver et al. [10] defined and referenced 25 terms for characterizing sensory properties of six Australian strawberry cultivars at two maturation stages (under-ripe and ideal maturation stage). Vázquez-Araújo et al. [11] developed 35 descriptors with definitions and references for describing flavor and texture characteristics of 20 pomegranate cultivars. A lexicon was developed for comparison of flavor and texture properties between fresh mangoes and heat-treated mango purées of six mango cultivars [12]. A lexicon for sweet tamarind, a major edible fruit and flavoring ingredient in Asia, was established based on six sweet tamarind cultivars [13]. The lexicon revealed 14 attributes that were present in all cultivars and seven attributes that provided uniqueness for some cultivars. Chambers et al. [14] developed a lexicon of 17 attributes that distinguished flavor and key texture characteristics of cabbage (*baechu*) kimchi (traditional Korean side dish) of wedge vs. sliced types and fresh vs. fermented types. The attribute terms were given in English and Korean. A lexicon for dried fig was developed by Haug et al. [15] and it contained 68 terms for describing interior and exterior appearance, aroma, flavor, and aftertaste of dried fig samples of different cultivars.

### 2.2. Grains and Nuts 

Miller and Chambers [16] examined seven black walnut cultivars and developed a lexicon that described flavor differences among cultivars. Lynch et al. [17] further expanded the lexicon of Miller and Chambers [16] by examining three additional black walnut cultivars. An additional flavor attribute (banana-like) was added to the lexicon, along with terms for describing appearance, aroma, and texture characteristics of black walnut samples. The lexicon for pecan included 20 terms with which to describe flavor differences between cultivars in raw and roasted forms [18]. Griffin et al. [19] established a lexicon containing 29 attributes that allowed for characterization of flavor and texture differences among various cashew-nut samples, including raw, oil-roasted, dry-roasted, skin-on, and rancid types. A lexicon for quinoa, a pseudo-cereal similar to amaranth and buckwheat, was developed using 21 commercial quinoa varieties, and it consisted of 27 terms for describing variations in color, aroma, flavor, and texture characteristics among quinoa varieties [20]. More recently, a lexicon for cooked spaghetti was developed using a large sample set of spaghetti from various countries. The lexicon listed 35 attributes, of which 19 were for texture; this indicated a wide variation in texture characteristics among cooked spaghetti samples [21]. 

### 2.3. Beverages

A lexicon consisting of 32 aroma/flavor, taste, and mouthfeel attributes was developed for freshly pressed and processed blueberry juice [22]. Kim et al. [23] identified, defined, and referenced 23 terms that described the flavor and mouthfeel of commercial orange juice products sold in Korea. Bhumiratana et al. [24] developed a lexicon consisting of 15 terms for describing aroma characteristics of coffee samples at each preparation step (green coffee bean, roasted coffee bean, ground coffee, and brewed coffee) as affected by coffee varieties and roasting levels. Sanchez et al. [25] identified, defined, and referenced 28 terms for describing the aroma, flavor, and aftertaste of brewed coffee of different coffee varieties and brewing methods. More recently, Chambers et al. [26] developed a lexicon containing a large set of 110 terms for describing the aroma and flavor of a wide range of brewed coffee samples. Their coffee lexicon was created based on the evaluation of 105 coffee samples from 14 countries around the world. The researchers also organized all attributes into a coffee tree for a better understanding of coffee characteristics. For hibiscus tea, samples including freshly prepared and ready-to-drink-infusions, syrups, concentrates, and instant tea were evaluated, and 21 descriptors were defined and referenced to describe the appearance, aroma/flavor, and mouthfeel of the samples [27]. All descriptors were also assembled into a sensory wheel. A lexicon for pink port wine was developed by Monteiro et al. [28] and it consisted of 21 descriptors with which to describe differences in appearance, aroma, flavor, mouthfeel, and aftertaste of the wine samples from five different brands. 

### 2.4. Bakery Products

Morais et al. [29] established a lexicon containing 15 terms for describing variations in the appearance, aroma, flavor, and texture of pre-biotic gluten-free bread formulated with different types and concentrations of sweeteners and prebiotics. Jervis et al. [30] developed a lexicon consisting of 36 terms that described the diversity in crust and crumb appearance, flavor, hand texture, and oral texture characteristics of whole-wheat sandwich bread. Cho et al. [31] defined and referenced 27 terms for characterizing the flavor and texture properties of *seolgitteok* (Korean rice cake) formulated with varying levels of brown rice flour and sugar.

### 2.5. Dairy Products

Newman et al. [32] developed a sensory lexicon for dairy protein hydrolysates produced from whey protein and casein substrates with varying degrees of hydrolysis. The lexicon consisted of 19 flavor attributes, among which bitter taste, metallic, and astringent were important characteristics for differentiating casein hydrolysates from whey protein hydrolysates. Brown and Chambers [33] established a lexicon that described flavor and texture differences among yogurt samples of varying milk sources (organic or conventional), percent milk fat and processing (set, stirred, or strained/Greek styles). A lexicon for artisan goat milk cheese was developed using 47 samples manufactured in different parts of the U.S., and it consisted of 39 flavor terms [34]. Twenty-eight of the terms were commonly present in goat milk cheeses and were able to describe most flavor characteristics of the samples. In addition, common attributes that described goat milk cheese of certain types (chèvre-style, feta-style, cheddar-style, and mold ripened type) were listed.

### 2.6. Soy Products

Recent examples of lexicons for soy products included those for soy sauce, soy milk, *sufu* (fermented soybean curd, a side-dish or condiment of traditional Chinese cuisine) and *doenjang* (Korean fermented soybean paste). Cherdchu et al. [35] examined 20 representative soy sauce samples produced from several Asian regions and the U.S., and developed a lexicon consisting of 58 terms for describing the diversity in flavor characteristics of the soy sauce category. Since the soy sauce lexicon was developed based on an agreement between U.S. and Thai descriptive panels, the attribute terms were given in both English and Thai. The soy sauce lexicon of Cherdchu et al. [35] was expanded further in two later studies. Pujchakarn et al. [1] developed a lexicon consisting of 50 attributes for describing the appearance, aroma, flavor, and aftertaste of seasoning soy sauce, a specific subcategory of soy sauce. Seasoning soy sauce is mainly produced by a chemical hydrolysis process with an addition of other ingredients such as sugar, vinegar, and flavor enhancers. Many terms in the seasoning soy sauce lexicon were found in the general soy sauce lexicon of Cherdchu et al. [35], while seven new flavor attributes (“dark fruity”, “celery”, “Chinese radish”, “fermented soybean”, “malt/cereal”, “musty”, “prickly”) were detected. Imamura [36] evaluated 149 soy sauce samples of varying manufacturing countries and methods and developed an exhaustive list of 88 terms for describing the aroma, flavor, and texture characteristics of the samples. The researchers also organized all attributes into a flavor wheel for a better understanding of the soy sauce characteristics. In addition, they reported that 19 out of the 88 attributes were common characteristics present in all soy sauce samples, and that evaluation of the 19 attributes was adequate for distinguishing naturally brewed soy sauce from chemically hydrolyzed soy sauce. Lawrence et al. [37] developed a lexicon for unflavored soymilk based on the evaluation of 26 commercial soymilk samples available in the U.S. market. The lexicon contained 24 terms, most of which described the flavor characteristics of soymilk. For *sufu*, lexicons were developed for the plain type [38] and red type [39], based on the evaluation of commercial *sufu* samples. The plain *sufu* lexicon consisted of 22 terms, including aroma, flavor, and texture attributes, among which “salty”, “moldy”, “alcohol-like”, “sesame-like”, and “cohesiveness”were deemed important for sample differentiation. The red *sufu* lexicon contained 15 descriptors for appearance, aroma, flavor, texture, and aftertaste characteristics. Kim et al. [40] defined and referenced 31 terms that described the appearance, aroma, flavor, texture, and aftertaste of *doenjang,* as affected by microbial communities present in the sample. 

### 2.7. Meat Products

Baker et al. [41] developed a lexicon for sensory evaluation of caviar. The lexicon consisted of 18 descriptors with which to describe differences in appearance, aroma, flavor, and texture among caviar samples harvested from sturgeon-fed varying diets. Kim et al. [42] identified and referenced 18 terms for describing the appearance and flavor of chicken stock of varying forms (i.e., cube, liquid, and powder). Samant et al. [43] developed a lexicon consisting of 28 terms for describing the effects of smoking and marination on the aroma, flavor, and texture characteristics of chicken breast fillets. Lexicons for some traditional meat products, such as *salama da sugo* (a typical fermented sausage produced in Italy), Lucanian dry-cured sausage (Italian pork-based sausage), *morcela de Arroz* (Portuguese cooked blood sausage), bulgogi (Korean barbecued beef), and *larou* (Chinese traditional bacon) were also published. Coloretti et al. [44] developed a lexicon for the sensory evaluation of *salama da sugo*. The lexicon consisted of 23 descriptors, including “wine aroma”, “spicy aroma”, “astringent”, “pricking”, “fat/lean connection”, and “fibrosity”, among others. The lexicon for Lucanian dry-cured sausage consisted of 21 terms that described sensory characteristics of the product, as affected by the presence or absence of nitrates and nitrites as curing agents [45]. For Portuguese cooked blood sausage,14 descriptors were defined and referenced [46]. The bulgogi lexicon listed 17 terms for describing the aroma, flavor, and texture of the product, as affected by varying levels of sugar, soy sauce, garlic, or sesame oil [47]. The terms were given in English and Korean. Wang et al. [48] identified and referenced 17 descriptors for describing a wide range of aroma and flavor characteristics of commercial *larou* samples produced from different geographic regions of China. The descriptors were given in English and Chinese.

### 2.8. Foods for Animals

To date, only two sensory lexicons for animal foods have been published in recent years, one for dog food and one for cat food. A lexicon for dry dog food was published by Di Donfrancesco et al. [49] who examined 21 representative dry dog food samples that were selected from approximately 200 samples commonly sold in the U.S., then defined and referenced 72 terms for describing the appearance, aroma, flavor, and texture of the samples. More recently, Koppel and Koppel [50] developed an aroma lexicon for retorted cat food. A total of 30 attributes were defined and referenced. The main aroma attributes in retorted cat food included “meaty”, “brothy”, “cooked”, “vitamin”, and “barnyard”, and depending on the ingredients used, also included “poultry”, “beefy liver”, “seafood”, and “heated oil” aromas. 

### 2.9. Miscellaneous Items 

Rosales et al. [51] defined and referenced 26 terms to describe the appearance, flavor, and texture of dark compound chocolate, as affected by the addition of crystal promoter additives. Jaffe et al. [52] explored smoky characteristics and developed a lexicon to describe the smoky flavor of a wide variety of smoked products, such as sausage, bacon, chicken, turkey, and ham lunch meat, marinade, barbeque sauce, cheese, fish, and liquid smoke. Fourteen attributes were defined and referenced, including aroma terms such as “smoky (overall)”, “ashy”, “woody”, “burnt”, “acrid”, “pungent”, “petroleum-like”, “creosote/tar”, “cedar”, and “bitter”, among others. Chambers et al. [53] defined and referenced 21 descriptors that described the texture properties of thickened foods during ingestion, swallowing, and after swallowing. The descriptors could be used to evaluate and compare the texture characteristics of thickened food products prescribed for patients with dysphagia. 

For ease of reference, Table 1 lists the recent lexicons mentioned above, with details including: (1) the number of samples being examined for lexicon development, (2) whether or not the definition, reference, and reference intensity are given in the lexicons, and (3) the number of descriptors listed in the lexicons. It is recommended that the number of samples being examined during lexicon development should cover the entire product space [2]. The appropriate size of the sample set depends on the diversity of the product category, as well as the objective of the study. For studies that aim to develop a lexicon for describing sensory differences among samples as affected by certain factors varying at certain levels, a not very large sample set could perhaps represent the entire product space. This was the case for some of the studies shown in Table 1, in which the lexicons were developed and subsequently used to determine samples’ sensory characteristics as affected by cultivars [9], cultivars/varieties and processing/preparation methods [12,24,25,43], and ingredients/additives [29,31,45,47,51] that were varied at limited levels. The number of samples being examined in those studies varied from 4 to 15. On the other hand, the rest of the studies in Table 1 aimed to develop general lexicons for specific product categories; therefore, a larger sample set was used in most studies in order to represent each entire product space. For instance, 105 coffee samples from countries around the world were examined to develop a general lexicon for brewed coffee [26]. Apple samples of 78 cultivars were used to develop a general lexicon for apple [6]. Forty retorted cat food samples varying in processing, ingredients, and packaging that represented the product market space were examined to develop a general lexicon for retorted cat food [50]. Twenty representative soy sauce samples produced in different countries were used to develop a general lexicon for soy sauce [35]. However, there were some studies [10,13,14,15,16,17,23,28,38,39,40,42,44,46] that used quite a small number of samples (*n* = 5–14) to develop a general lexicon for a specific product category. Those few samples were less likely to be able to fairly represent the entire product space. Consequently, the lexicons developed in those studies should be considered as “initial” lexicons, meaning that more attributes may become apparent in future studies with more varieties of samples.

When a large number of samples are used to develop a lexicon, samples are often placed in different sets to be used at different phases of lexicon development. For example, Chambers et al. [26] placed 105 coffee samples in four sets (*n*=13, 45, 27, 20 for sets 1–4, respectively). Set 1, with a narrow range of coffee samples, was used for initial lexicon development. Subsequently, Set 2, with an array of commercial coffee samples, and Set 3, with samples having unique sensory characteristics, were used to expand the lexicon and incorporate additional attributes. Lastly, Set 4, with a narrow range of coffee samples, was used to validate that the developed lexicon could explain diverse sensory characteristics present in all samples and, at the same time, could detect small differences among the samples. Another approach of sample arrangement was adopted in a study of Cherdchu et al. [35] in which a total of 116 soy sauce samples were procured from different manufacturing regions and initially screened by sensory analysts; thereafter, only 20 samples that represented the diversity in flavor characteristics of soy sauce were used for developing a soy sauce lexicon. 

It should be noted that definitions and character references were provided for each descriptor in almost all of the lexicons listed in Table 1. According to Drake and Civille [4], the definition and character reference are crucial components of a sensory lexicon, since they help to maximize language clarity and enable panelists and other audiences to clearly understand the concept of each term. However, there was one study [32] that only provided the character references without a term definition. Most of the lexicons in Table 1 also provided the intensity of each reference to anchor an attribute scale that could help reduce panel variation in the intensity rating. 

## 3. Applications of Lexicons

Applications of sensory lexicons as an effective communication tool and a guidance tool for research and development of food products are as follows.

### 3.1. Communication Tool

Lexicons facilitate accurate and precise communication regarding products’ sensory characteristics across diverse audiences, such as sensory panels and scientists, product developers, marketing professionals, and suppliers, both within and between companies and even between countries [2,21]. Different people, especially those from different countries and cultures, may have a different understanding and interpretation of the same descriptor. However, this deviation could possibly be solved with the use of a well-developed lexicon in which definition and character reference(s) for each sensory descriptor are provided. For example, Cherdchu et al. [35] found that U.S. and Thai panels had difficulties understanding some of the other panel’s terms for describing the flavor characteristics of soy sauce. Thai panelists had difficulty differentiating the terms “brown”, “caramel”, and “dark brown” that were used by U.S. panelists. Both panels had difficulties differentiating the terms “earthy-damp”, “dusty”, and “moldy-damp”. However, the problems were solved once the meaning of each term was clearly defined with an agreement of both panels and an appropriate reference standard was used to represent each term. This study highlighted the importance of term definitions and reference standards for cross-cultural sensory research. A study by Vázquez-Araújo et al. [54] demonstrated that a lexicon for *turrón* (European confectionary product) that was developed by the U.S. panel could be adopted effectively by the Spanish panel for evaluating the product. They found that flavor attributes of *turrón* samples were rated in the same way by both panels, suggesting a similar understanding toward flavor attributes of the two panels. However, there were inconsistencies between the two panels on some texture attributes. The authors suggested that definitions and references of these texture attributes should be adapted. 

Some terms describe complex attributes which may exert a different character depending on products in which they are present. For example, both spinach and cucumber could be described as “green”; however, the “green” character of spinach is different from that of cucumber. Therefore, it would be advantageous if ones could describe any products using more specific terms. Hongsoongnern and Chambers [55] explored the “green” character of various food categories. They found that the “green”character could be described using five terms: “green-unripe”, “green-peapod”, “green-grassy/leafy”, “green-viney”, and “green-fruity”, and food examples of these green related terms were green banana, raw peanut, spinach, cucumber, and green apple, respectively. In addition to the “green” attribute, research had been carried out to develop lexicons for describing other complex sensory attributes, including “smoky” [52], “nutty” [56], and “beany” [57,58]. This style of lexicon could be useful for communication among various audiences.

### 3.2. New Product Development

For the food industry, new product development is one of their most important activities. The process of developing new products can be divided into several stages, among which concept development, prototyping, and commercialization are critical steps to the success of a new product [59]. Descriptive tests and sensory lexicons developed by trained panelists could be used as effective guidance tools in a new product development process as follows.

#### 3.2.1. Concept Development

For concept development, information is gathered from the literature, patents, market trends, and competitive products in the market. Descriptive sensory analysis could provide better understanding of sensory properties of existing or competitive products where the potential new product will be placed [60]. A number of lexicons were developed and used to determine sensory profiles of commercial products, such as cabbage kimchi [14], hibiscus tea [27], goat milk cheese [34], French cheese [61], pomegranate juice [62], green tea [63], plain *sufu* (Chinese fermented soybean curd) [38], dry dog food [49], and retorted cat food [50], among others. 

In addition, combining descriptive data with consumer acceptability data could assist in identifying which products are liked for which reason, and possibly providing insight on potential gaps in the market [60]. For instance, Lawrence et al. [37] studied sensory profiles of 26 commercial plain soymilks available in the U.S. market using a lexicon consisting of 24 terms, and subsequently selected 12 representative soymilk samples based on their sensory profiles for testing with consumers from different age/cultural categories. Based on liking data, three consumer clusters were identified. Consumers in Cluster 1 were mainly Asian females aged 18–30 years, while those in Cluster 2 were mainly Caucasian/African American females aged 40–64 years. Cluster 3 consisted of Caucasian/African American females aged 18–30 and 40–64 years. With a preference mapping technique, the drivers of liking for each consumer cluster were identified. Sweet taste, sweet aromatic, vanilla/vanillin flavor, and higher viscosity were preferred by all consumer clusters, and differences among the clusters were the drivers of disliking. There was no driver of disliking identified for Cluster 1, while Clusters 2 and 3 disliked soymilk with “beany”, “green/grassy”, and “meaty/brothy” flavors and astringency. The consumers in Cluster 3 were willing to overlook disliked attributes with the addition of sweet taste, while those in Cluster 2 were not. 

In another study, Lykomitros et al. [64] reported that roasted peanut, dark roast and sweet aromas, and sweet taste were the drivers of liking, while raw bean aroma and bitter taste were detractors for the liking of roasted peanut for European consumers. Kim et al. [23] studied sensory profiles of commercial orange juice products in the Korean market using a lexicon with 23 descriptors, and identified drivers of liking of Korean consumers. They found three consumer segments based on liking. Consumers in Segment 1 (46%) preferred orange juice products with strong orange flavor, which could be either artificial or natural. Consumers in Segment 2 (29%) were inclined to “functional flavors”, such as “sour”, “bitter”, and “medicinal”, while those in Segment 3 (25%) liked orange juice products that were high in sweet taste but low in sour and bitter tastes.

Studies on drivers of liking and disliking of other food products, such as gluten-free bread [29], whole wheat sandwich bread [30], *doenjang* (Korean fermented soybean paste) [40], caviar [41], and sweet potato [65] were also available in the literature. An understanding of how sensory properties affect consumer preferences is very important to product developers to create products with attributes that are well-liked by target consumers and to tailor attributes for segments of the population that have not yet been accommodated [37]. A profile of desirable sensory characteristics not only aids the development of products, but also serves as a reference guide once prototypes are being developed [60].

#### 3.2.2. Prototyping

Carrying out descriptive analyses using sensory lexicons throughout the development stage is advantageous for product developers, as it helps them to understand the effects of ingredients, processing methods, packaging conditions, etc. on the sensory qualities of products in detail. In addition, the developed products should be compared with desirable sensory characteristics identified in the product concept [60]. For instance, Cho et al. [31] used a lexicon consisting of 27 descriptors to determine the effects of the amount of brown rice flour and sugar on flavor and texture properties of *seolgitteok* (Korean rice cake). The *bulgogi* lexicon, with five aroma, ten flavor, and two texture terms was used to assess the effects of sugar, soy sauce, garlic, and sesame oil on the sensory characteristics of the product [47]. A lexicon developed for dairy protein hydrolysates (casein and whey protein hydrolysates) could be useful for developing products using these ingredients as protein sources, such as baby foods, nutrient-rich beverages, sport drinks, etc. [32]. In particular, attention should be paid to the attributes in the lexicon—namely, “sulfurous”, “wet dog/animal”, “metallic”, “bitter”, and “astringent”, since they are undesirable characteristics caused by dairy protein hydrolysates. Ledeker et al. [66] conducted a descriptive test using a lexicon with 46 descriptors to understand the effects of heating on the flavor and texture qualities of mango purée and sorbet, prepared from four mango cultivars available in the U.S. Later, a similar study was conducted on mango purée prepared from Thai mango cultivars [12]. Jáuregui et al. [67] used a lexicon containing 13 descriptors to understand the effects of different vegetable oils on the sensory quality and stability of fried salted soybean. 

Once the product prototype is developed, a sensory lexicon could be used for comparing the sensory property of the prototype against that of commercial products, especially the category leaders, to ensure that the prototype will be successful in the market. For example, Brown and Chambers [33] compared the sensory characteristics of two prototype yogurts against 26 commercial plain yogurts. The prototypes differed from commercial plain yogurts in that they had undergone an additional pre-acidification step, in which milk was pre-acidified with lemon juice or citric acid to pH 6.2 prior to the normal fermentation process in order to shorten fermentation time and thus reduce production energy. Highly trained descriptive panelists evaluated all yogurt samples using a lexicon consisting of 35 attributes. Results showed that flavor and texture characteristics of the prototypes were comparable to those of leading brands, thereby suggesting potential market viability of the prototypes.

#### 3.2.3. Commercialization

Product commercialization is a full scale-up and integration of production and marketing [59]. Scaling-up from the test kitchen to the pilot plant to the factory trail always produces changes in a product. Performing descriptive tests using a lexicon could assist in understanding subtle sensory changes during a scale-up, and could help find solutions for bringing the product back to the originally intended sensory characteristics [60].

### 3.3. Quality Control

The role of sensory evaluation in quality control usually involves the maintenance of consistency of food quality [60]. Descriptive sensory analysis and lexicons could be used to determine the target specification of food products in terms of sensory quality, and to test if the product complies with the target specification [68]. In setting quality specifications, the relationship between consumer acceptance and descriptive panel responses should be established, and a method to determine such a relationship was described in detail by Moskowitz et al. [69]. A study by Chambers et al. [14] demonstrated a possible use of lexicons for quality control purposes. In their study, a lexicon consisting of 17 terms were used to evaluate flavor and texture characteristics of Korean commercial cabbage kimchi (including wedge vs. sliced types, and fresh vs. fermented types). Results showed that there were products of the same type from the same manufacturer with totally different sensory characteristics. Since they were commercial products, it was not known with certainty whether that deviation was due to a poor quality-control system, or whether it was because the manufacturer had intentionally different products to appeal to different segments of the consumer market. In the case where an improved quality-control system was needed, the lexicon developed in the study could be used to pinpoint different characteristics and then adjust the production process to improve product consistency. 

### 3.4. Product Improvement

Descriptive sensory analyses and lexicons could be used to monitor whether changes in ingredients, processing methods, and packaging could improve the quality of a company’s existing products. For example, a study by Rosales et al. [51] used a descriptive test and a lexicon containing 25 well-defined and referenced terms to determine if heat-resistant properties and the sensory quality of dark-compound chocolate could be improved with the addition of a crystal-promoter additive. Three crystal-promoter additives were tested at varying concentrations. Results showed that the additive CP1 (at 0.25% level) which was composed of mono- and diglycerides and polyglycerol esters from high oleic sunflower oil was better than the other two additives at all concentration levels, resulting in compound chocolate with high heat-resistant properties and with higher cocoa, dark brown, bitter aromatic, and sweet intensities, as well as a faster melting rate and less wax-coating mouthfeel.

### 3.5. Measuring Changes during Product Shelf-Life

ASTM International [70] defined sensory shelf-life as “the time period during which the products’ sensory characteristics and performance are as intended by the manufacturer”. Descriptive tests and lexicons developed by trained panelists are effective tools in shelf-life studies. Such tools could be used to track changes in the sensory characteristics of a product over time, or to determine how long a product can be stored before there is a noticeable change in sensory quality [68]. 

For instance, Rosales and Suwonsichon [7] monitored changes in the sensory quality of five pomelo cultivars during seven-day fresh-cut storage (4 °C, 85% relative humidity) using a lexicon consisting of 30 attributes established by a highly trained descriptive panel. They found that pomelo samples of Tubtim Siam, Thong Dee, and Kao Numpeung cultivars showed only minimal changes throughout the storage period. While the Kao Tangkwa cultivar showed noticeable decreases in hardness, firmness, chewiness, and sourness, it showed an increase in pomelo identity and overall sweet notes after storage for three days. For Kao Yai cultivar, changes in sensory quality became more evident after storage for five days, resulting in increased hardness and firmness but decreased sweetness. Such information could be useful for retailers in regard to knowing the approximate storage duration of the pre-cut pomelo fruit of different cultivars without noticeable quality changes. 

Riveros et al. [71] compared sensory profiles of peanut paste prepared from high-oleic and normal peanuts. A lexicon consisting of 16 attributes was developed for tracking the sensory changes of the samples during storage at 4, 23, and 40 °C for up to 175 days. Results showed significant changes in three attributes, with peanut paste prepared from high-oleic peanut having increases in oxidized and cardboard flavors and a decrease in roasted peanut flavor at slower rates than that prepared from normal peanuts. In another study of the same researchers, changes in sensory attributes of roasted peanuts coated with different edible coatings (carboxymethyl cellulose, methyl cellulose, and whey protein) were monitored during storage at 40 °C for up to 56 days [72]. Trained panelists rated intensities of 13 attributes selected from the peanut lexicon of Johnsen et al. [73], three of which changed significantly during storage. Roasted peanut flavor was lower, while oxidized and cardboard flavors were higher for uncoated peanuts than for coated ones. Among the edible coatings, carboxymethyl cellulose provided the best protection effect. 

Lee and Chambers [74] determined changes in flavor characteristics of two Korean green teas during storage at 20 °C for 3, 6, 12, 18, and 24 months. Twenty flavor attributes selected from the green tea lexicon developed earlier by the same authors [75] were evaluated. At each storage period, panelists rated attribute intensities of the samples by comparing them to the reference standard scores provided in the lexicon. The authors pointed out that comparison to reference standards was necessary to ensure that the panelists did not drift in their assessments over the two-year testing period. This study highlighted the importance of quantitative references in the lexicon for long-term storage testing. Results revealed that both green tea samples changed only slightly over a period of two years. 

### 3.6. Breeding New Plant Cultivars 

Plant breeding has been practiced for thousands of years to change the traits of plants in order to produce the desired characteristics [76]. Understanding sensory characteristics and consumer preference is an important key to develop and select novel plant cultivars that will be successful in the marketplace [6]. Lexicons have been widely used for descriptive tests to identify, quantify, and compare sensory characteristics of fruits and vegetables among cultivars and to relate descriptive data to instrumental data and consumer liking to aid in breeding new successful cultivars. Lexicons established for fruits, vegetables, and nuts which could be used for this purpose are prominent in the literature, some of which are shown in Table 1. 

The application of sensory lexicons in plant breeding can be demonstrated through the studies of Bowen et al. [6], Du et al. [9], and Vara-Ubol et al. [77]. Bowen et al. [6] determined sensory profiles of 78 apple cultivars using a lexicon established by a trained descriptive panel. Nineteen representative apple cultivars were then subjected to an acceptance test using 219 consumers. Afterward, relationships between sensory profile data and liking data were determined through the external preference mapping technique. Results showed that apple cultivars could be classified into four groups based on the following flavor and texture properties: “aromatic-sweet”, “acidic”, “mealy”, and “balanced”. Most consumers (89%) preferred apple cultivars that were sweet and which had a fresh red-apple aroma, while the rest (11%) liked apple cultivars that had an acidic taste and green-apple aroma. For all consumers, a high level of perceivable crispy and juicy texture was a strong driver of liking, whereas a mealy texture was a strong driver of disliking. Such information, as well as an external preference map created in this study, could be useful for selecting apple cultivars with desired sensory characteristics for crossing in a breeding program. 

In Du et al.’s study [9], flavor characteristics of eight thornless blackberry cultivars were compared against those of Marion, a thorny cultivar. Although Marion is the most commonly marketed cultivar, its thorns can be dangerous to consumers if they end up in the fruit. Therefore, the aim of the study was to identify thornless cultivars that had similar or superior flavor quality to the Marion cultivar. The thornless blackberry cultivars being tested included six commercial cultivars (Thornless Evergreen, Black Diamond, Black Pearl, Nightfall, Waldo, and Chester) and two newly bred cultivars (ORUS 1843-3 and NZ 9351-4). Trained descriptive panelists evaluated all of the blackberry cultivars using a lexicon consisting of 17 flavor attributes. Results showed that four (Black Diamond, Black Pearl, ORUS 1843-3, and NZ 9351-4) out of eight thornless cultivars were similar to the Marion, having high intensities of “fresh-fruity”, “raspberry”, “floral”, and “strawberry” flavors, while the rest of the thornless cultivars had high intensities of“vegetal”, “woody”, “moldy”, and “cooked fruit” flavors. Results of this study could be provided as a guide to blackberry breeding programs to develop or select thornless cultivars with superior fruit quality.

Vara-Ubol et al. [77] determined sensory characteristics of eight rose-apple varieties cultivated in Thailand, five of which (Pet-Num-Ping, Toon-Klao, Tub-Tim-Jun, Pet-Sai-Rung, and Pet-Sam-Pran) were popular in the marketplace, while three of which (Ma-Meow, Num-Dok-Mai, and Sa-Rak) were not. Results showed distinct differences in sensory characteristics between the popular and unpopular cultivars. The former had a crispy texture but bland flavor, while the latter had a spongy, mealy, and firm texture with strong fruity, floral/perfumy, rose, and woody flavor notes. The authors pointed out the possibility that the cultivars Pet-Num-Ping, Toon-Klao, Tub-Tim-Jun, Pet-Sai-Rung, and Pet-Sam-Pran could become even more accepted by consumers if plant breeders could combine the crispy texture of the cultivars with the rose and floral flavor notes.

## 4. Conclusions

A number of recent sensory lexicons developed by trained panelists for documenting and describing sensory characteristics of various food products have been provided in the current review. This review has also shown how the lexicons can be used as an effective communication tool and research guidance tool for new product development processes, quality control, product improvement, measuring changes of products during storage, and breeding new plant cultivars. 

## Figures and Tables

**Table 1:** Examples of recent food lexicons.

	Number of Samples Used in Lexicon Development	Definitions	Character References	Reference Intensities	Numbers of Attributes in the Lexicon					Source, Published Date
					Appearance	Aroma	Flavor	Texture/Mouthfeel	Aftertaste	
**Fruits and Vegetables**										
Apple	40	Yes	Yes	Yes	2	1	5	7	-	[5], 2013
Apple	78	Yes	Yes	No	-	-	12	6	-	[6], 2018
Pomelo	20	Yes	Yes	Yes	1	7	10	9	3	[7], 2015
Peach	51	Yes	Yes	Yes	1	11	14	7	-	[8], 2017
Blackberry	9	Yes	Yes *	No	-	17	-	-	-	[9], 2010
Strawberry	12	Yes	Yes	No	2	8	10	5	-	[10], 2018
Pomegranate	20	Yes	Yes	Yes	1		26	8	-	[11], 2014
Mango	12	Yes	Yes	Yes	-	-	30	10	-	[12], 2014
Sweet tamarind	12	Yes	Yes	Yes	-	-	17	9	-	[13], 2010
Cabbage kimchi	14	Yes	Yes	Yes	-	-	15	2	-	[14], 2012
Dried fig	12	No	Yes	No	13	20	23	7	5	[15], 2013
**Grains and nuts**										
Black walnut	7	Yes	Yes	Yes	-	-	22	-	-	[16], 2013
Black walnut	10	Yes	Yes	Yes	3	8	23	6	-	[17], 2016
Pecan	32	Yes	Yes	Yes	-	-	20	-	-	[18], 2016
Cashew nut	15	Yes	Yes	No	-	-	25	4		[19], 2017
Quinoa	21	Yes	Yes	Yes	3	9	7	8	-	[20], 2017
Spaghetti	50	Yes	Yes	Yes	5	9	2	19	-	[21], 2018
**Beverages**										
Blueberry juice	20	Yes	Yes	No	-	27 (aroma/flavor)		5	-	[22], 2013
Orange juice	7	Yes	Yes	No	-	-	15	8	-	[23], 2013
Coffee	9	Yes	Yes	Yes	-	15	-	-	-	[24], 2011
Brewed coffee	12	Yes	Yes	Yes	-	10	15	-	3	[25], 2015
Brewed coffee	105	Yes	Yes	Yes	-	110 (aroma and flavor)		-	-	[26], 2016
Hibiscus tea	22	Yes	Yes	No	4	15 (aroma and flavor)		2	-	[27], 2017
Pink port wine	5	Yes	Yes	Yes	3	7	6	4	1	[28], 2014
**Bakery products**										
Prebiotic gluten free bread	6	Yes	Yes	Yes	3	4	3	5	-	[29], 2014
Whole wheat sandwich bread	25	Yes	Yes	Yes *	5	-	19	12	-	[30], 2016
*Seolgitteok* (Korean rice cake)	15	Yes	Yes	Yes	-	-	12	15	-	[31], 2014
**Dairy products**										
Dairy protein hydrolysate	25	No	Yes	Yes	-	-	19	-	-	[32], 2014
Yogurt	29	Yes	Yes	Yes	-	-	25	10	-	[33], 2015
Goat milk cheese	47	Yes	Yes	Yes	-	-	39	-	-	[34], 2016
**Soy products**										
Soy sauce	20	Yes	Yes	Yes	-	-	58	-	-	[35], 2013
Seasoning soy sauce	25	Yes	Yes	Yes	1	22	24	-	3	[1], 2016
Soy sauce	149	Yes	Yes	No	-	47	37	4	-	[36], 2016
Soymilk	26	Yes	Yes	Yes *	1	1	21	1	-	[37], 2016
Plain *sufu* (Chinese fermented soybean curd)	12	Yes	Yes	Yes		8	7	7	-	[38], 2016
Red *sufu* (Chinese fermented soybean curd)	12	Yes	Yes	Yes	1	4	5	4	1	[39], 2018
*Doenjang* (Korean fermented soybean paste)	14	Yes	Yes	No	3	8	10	5	5	[40], 2016
**Meat products**										
Caviar	55	Yes	Yes	Yes	6	1	7	4	-	[41], 2014
Chicken stock	10	Yes	Yes	Yes	5	-	13	-	-	[42], 2017
Chicken breast filet	4	Yes	Yes	Yes	-	10	12	6	-	[43], 2016
*Salana da sugo* (Italian fermented sausage)	12	Yes	Yes	Yes	2	3	10	8	-	[44], 2015
Lucanian dry sausage (Italian pork-based sausage)	10	Yes	Yes	Yes	7	4	6	4	-	[45], 2016
*Morcela de Arroz* (Portuguese cooked blood sausage)	12	Yes	Yes	Yes	4	3	3	4	-	[46], 2015
*Bulgogi* (Korean traditional barbecued beef)	4	Yes	Yes	No	-	5	10	2	-	[47], 2011
*Larou* (Chinese traditional bacon)	24	Yes	Yes	Yes	-	17 (aroma/flavor)		-	-	[48], 2018
**Animal foods**										
Dry dog food	21	Yes	Yes	Yes	16	44 (aroma/flavor)		12	-	[49], 2012
Retorted cat food	40	Yes	Yes	Yes	-	30	-	-	-	[50], 2018
**Miscellaneous**										
Compound chocolate	8	Yes	Yes	Yes	2	-	13	11	-	[51], 2018
Smoke attribute	54	Yes	Yes	Yes	-	-	14	-	-	[52], 2017
Thickened liquids	40	Yes	Yes	Yes	-	-	-	21	-	[53], 2017

* Reference intensities are given for some attributes.
